# Diagnostic value of the *UCA1* test for bladder cancer detection: a clinical study

**DOI:** 10.1186/s40064-015-1092-6

**Published:** 2015-07-16

**Authors:** Dina Milowich, Marie Le Mercier, Nancy De Neve, Flavienne Sandras, Thierry Roumeguere, Christine Decaestecker, Isabelle Salmon, Sandrine Rorive

**Affiliations:** Department of Pathology, Erasme University Hospital, Université Libre de Bruxelles, 808 Route de Lennik, 1070 Brussels, Belgium; Department of Pathology, Jules Bordet Institute, Université Libre de Bruxelles, Brussels, Belgium; Department of Urology, Erasme University Hospital, Université Libre de Bruxelles, Brussels, Belgium; Laboratory of Image Synthesis and Analysis (LISA), Brussels School of Engineering/École Polytechnique de Bruxelles, Université Libre de Bruxelles, Brussels, Belgium; DIAPath, Center for Microscopy and Molecular Imaging (CMMI), Académie Universitaire Wallonie-Bruxelles, Gosselies, Belgium

**Keywords:** *UCA1* RNA, human, *Urothelial carcinoma associated 1*, Bladder cancer, Biomarker, Urinary marker

## Abstract

**Purpose:**

To evaluate the efficiency of the *UCA1* test as a diagnostic tool for the detection of bladder cancer.

**Methods:**

Between October 2009 and December 2011 the *UCA1* test was performed on collected urine samples from 162 patients divided into screening and follow-up groups, based on the absence or presence of prior bladder cancer. The test performance was then evaluated in each group and compared to cystoscopy and urinary cytology.

**Results:**

The overall sensitivity, specificity and positive and negative predictive values for the *UCA1* test were 70, 70.7, 75.6 and 64.5%, respectively. We observed no difference in performance for tumours of higher grade or stage, but sensitivity was increased in the screening population compared to patients under follow-up (83.9 vs. 59%). The *UCA1* test successfully detected all 7 cases of isolated carcinoma in situ and was more sensitive in this particular setting than cystoscopy or urinary cytology.

**Conclusion:**

The efficiency of the *UCA1* test for the detection of primary and recurring bladder cancer in our study was lower than previously reported. We confirmed the role of *UCA1* as a possible adjunct to cystoscopy and cytology when a primary bladder cancer is suspected, but its role in the follow-up of recurring tumours remains limited. Further studies are needed to investigate the role of the *UCA1* test in the early detection of carcinoma in situ lesions.

## Background

Bladder cancer (BC) is the second most common urologic cancer. Although the majority of cases are diagnosed at early stages, up to 50% of tumours recur and 15–40% grow into muscle invasive disease (Amin et al. 2015). According to current guidelines, the diagnostic standard for BC is cystoscopy, often combined with urinary cytology (Kamat et al. 2013). This is also the approach used to follow-up patients with a history of BC. Though photodynamic diagnosis has improved the sensitivity of white cystoscopy for carcinoma in situ (CIS) lesions, both procedures remain invasive, time-consuming and costly, therefore making BC one of the most expensive malignancies to monitor and treat (Isfoss 2011; Van Rhijn et al. 2009). Cytology is a non-invasive test often performed on voided urine, with high specificity (96%) but low sensitivity (44%), particularly for low-grade tumours (Kamat et al. 2013). Several urine markers have been studied to help diagnose BC, and thereby decrease the need for cystoscopy as well as make following-up bladder cancer patients more cost-effective. To date, the Food and Drug Administration has approved six urine biomarkers for BC detection (Kamat et al. 2013; Tilki et al. 2011). Most markers have shown better sensitivity compared to cytology; however, their specificity remains lower (Tilki et al. 2011). None of those markers are currently recommended as standard diagnostic tools in routine urology (Kamat et al. 2013; Tilki et al. 2011).

*Urothelial carcinoma associated 1* (*UCA1)* was recently identified as a non-coding RNA upregulated in BC compared to normal bladder tissues, and is thought to be involved in embryogenesis and in BC progression (Wang et al. 2006, 2008, 2012). Overexpression of *UCA1* in the BLS-211 BC cell line significantly enhanced the tumorigenicity, invasion potential and drug resistance, both in vitro and in vivo (Wang et al. 2008). Yang et al. showed that *UCA1* stimulates cell proliferation via the p300 coactivator CREB, through a PI3K-AKT dependent pathway (Yang et al. 2012). *UCA1* was also identified as a very sensitive and specific urine marker of BC (Wang et al. 2006; Zhang et al. 2012; Srivastava et al. 2014). A recent study yielded a sensitivity of 79.49% and a specificity of 79.73%, suggesting that the *UCA1* test could be used as an adjunct to cytology in early diagnosis of primary urinary BC (Srivastava et al. 2014). Those promising results motivated us to study the *UCA1* test in an independent cohort, with a view to validating *UCA1* as a reliable biomarker for BC detection. Instead of using fresh urine samples stored on ice, as previously described, we developed the *UCA1* test on fixed urine samples in order to facilitate its use in daily practice (Wang et al. 2006). The test has been accredited (BELAC, ISO15189) since September 2009. The present work aims to report our experience regarding the clinical value of the *UCA1* test when compared to routine diagnostic methods.

## Methods

### Patient selection

Freshly voided urine samples were obtained from 162 patients between October 2009 and December 2012 at Erasme University Hospital, after approval by the local Ethical Committee (Ref: P2010/338). Patients were included in the study if they were (1) evaluated for suspected primary BC, (2) under surveillance for BC or (3) followed for other urological conditions. The clinical data collected for each patient include age, prior medical history and treatment, symptoms, cystoscopic and/or imaging findings, and follow-up through August 2014 (Table 1). All BC diagnoses were confirmed by histology, except for one patient. This person’s histological confirmation was forgone due to advanced age, despite a visible bladder tumour at both cystoscopy and CT scan, as well as malignant urothelial cells identified by cytology. White cystoscopy was performed for all patients and regarded as positive in cases involving apparent papillary or flat lesions and considered negative if findings were normal.

**Table 1:** Patients demographics and baseline features

Overall patients (n = 161)	Screening group		Follow-up group		p value
	n = 79		n = 83		
Clinical features^a^					
Age (median (range))	68 (32–90)		70 (35–90)		
Hematuria	48	60.8%	5	6%	p < 0.00001
Urinary tract infection	8	10.1%	1	1.2%	p = 0.01
Benign prostatic hyperplasia	18	22.8%	6	7.2%	p = 0.004
Renal transplant	10	12.7%	5	6%	p = 0.1
Lithiasis	5	6.3%	1	1.2%	p = 0.09
Hydronephrosis	7	8.9%	2	2.4%	p = 0.07
LUTS	12	15.2%	1	1.2%	p = 0.0009
Schistozomiasis	2	2.5%	0	0%	p = 0.24
Aristocholic acid nephropathy	5	6.3%	5	6%	p = 1
Other malignancies	14	17.7%	15	18.1%	p = 1
*UCA1* test					p = 0.04
Positive	43	54.4%	33	39.8%	
Negative	36	45.6%	50	60.2%	
Cytology					p = 0.09
Positive	21	26.6%	14	16.9%	
Negative	49	62%	59	71.1%	
Unsatisfactory	9	11.4%	10	12%	
Cystoscopy					p = 0.56
Positive	31	39.2%	36	43.4%	
Negative	39	49.4%	45	54.2%	
Not performed	9	11.4%	2	2.4%	
Histology					p = 0.56
Benign	12	15.2%	15	18.1%	
Malignant	30	38%	39	47%	
Urothelial carcinoma	27		38		
Squamous carcinoma	2		0		
Adenocarcinoma	0		1		
Small cell carcinoma	1		0		
Not performed	37	46.8%	29	34.9%	
Stage					
pTis	1	1.3%	6	7.2%	
pTa	6	7.6%	15	18%	
pT1	9	11.8%	6	7.2%	
pT2	9	11.8%	8	9.6%	
pT3	4	5.1%	1	1.2%	
pT4	1	1.3%	3	3.6%	
Grade					
PUNLMP	–		–		
Low grade	7	8.9%	16	19.3%	
High grade	19	24%	16	19.3%	
CIS	1	1.3%	6	7.2%	
Not available	3	3.8%	–		
Multicentricity					
Single tumours	16	20.2%	20	24.1%	
Multiple tumours	10	12.6%	11	13.6%	
Not available	4	5.1%	1	1.2%	

^a^Some patients present multiple clinical features.

### Urine and tissue samples

Urine samples were collected in a tube containing 25 ml of the fixative solution Saccomanno™ (Prosan, Merelbeke, Belgium) and were delivered to the laboratory within 24 h. From these samples, 50 ml was used to conduct routine cytology. All cytological diagnoses were reviewed by a uropathologist (SR). Cytology was categorized as positive if cancer cells or cells with atypical changes suggesting malignancy were found, and as negative if normal cells or cells with inflammatory atypical changes were found.

**Figure 1:**
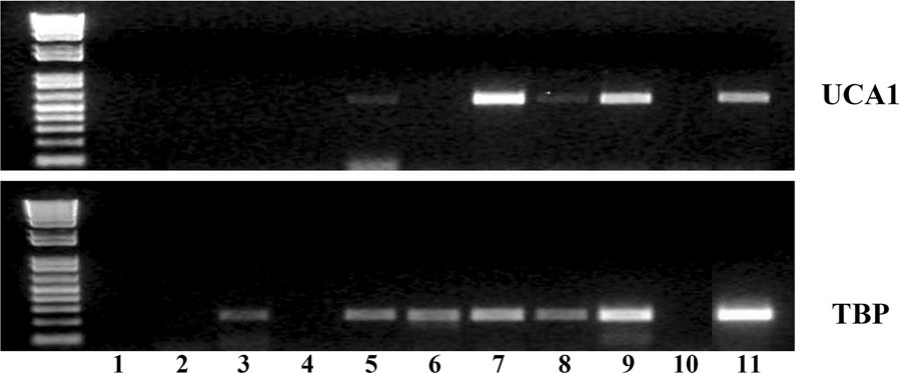
Expression of the UCA1 gene in urinary samples determined by semi-quantitative RT-PCR. This figure illustrates the expression of the UCA1 gene in urinary samples determined by semi-quantitative RT-PCR. Lanes 1 and 2 were negative controls for PCR (1) and reverse transcription (2). Lane 11 was the positive control consisting of RNA extracted from frozen bladder cancer tissue. TBP was used as an internal control. Cases (4) and (10) were considered unsatisfactory for evaluation because no TBP expression was detected. Cases (3) and (6) had no UCA1 expression, cases (5) and (8) had weak UCA1 expression and cases (7) and (9) had strong UCA1 expression.

The remaining urine volume was centrifuged at 3,000 rpm for 30 min and rinsed with PBS. The resulting pellets were suspended in RNAlater™ solution (Qiagen, Venlo, Netherlands) and stored at 4°C. RNA extraction was conducted using an RNeasy Mini kit (Qiagen) following the manufacturer’s recommendations. After checking the RNA quality and purity (NanoDrop 2000, Thermo Scientific, Aalst, Belgium), 50 ng of RNA extracts were submitted to reverse transcription using the Sensiscript Reverse Transcription Kit (Qiagen) and oligo(dT) primers (Invitrogen, Gent, Belgium). Amplification was performed using *UCA1* specific primers (Forward: 5′-GGGACTCCTTCGTGAGACC-3′ and Reverse: 5′-AGAGGAACGGATGAAGCCTG-3′). The *Tata box binding protein* (*TBP*) was used as a housekeeping gene (Forward: 5′-GGCACCACTCCACTGTATC-3′ and Reverse: 5′-AATCAGTGCCGTGGTTCGT-3′). For both PCR reactions, 40 cycles were run (Thermocycler T3, 94°C for 45 s, 57°C for 1 min, 72°C for 30 s). The PCR product was then resolved on agarose gels. Satisfactory tests were categorized using a three-tier score (negative, low or high *UCA1* gene expression). The test was considered positive if the *UCA1* gene was highly expressed, and negative if the *UCA1* gene was weakly or not expressed at all in the sample analysed (Figure 1). Two independent investigators (MLM and SR), blinded to the clinical data, cystoscopy findings and cytological diagnoses, evaluated the result of each *UCA1* test.

In 96 patients, tissue samples were retrieved by biopsy, transurethral resection of the bladder (TURB) or surgical procedures. The histopathological diagnoses were reviewed by a uropathologist (SR).

### Data analysis

Patients were divided into screening and follow-up groups, based on absence or presence of prior BC. Patients were considered disease-free if the workup was negative and no evidence of BC was found in the six following months. Patients with insufficient follow-up were excluded from the study. We evaluated the sensitivity, specificity, positive and negative predictive values of the *UCA1* test in both patient groups compared to cystoscopy and cytology.

## Results

Among the 162 patients included in this study, 79 had no prior history of BC and 83 were part of the follow-up cohort. For each of these two groups, the breakdown between patients in terms of clinical features, cytology, cystoscopy and *UCA1* test results is shown in Table 1. In all, 69 diagnoses of BC were made, of which 30 were primary BCs and 39 recurrent cases. Most cases (n = 65) were diagnosed as urothelial carcinomas, though two cases of pure squamous cell carcinoma and single cases of primary bladder adenocarcinoma and small cell carcinoma were also encountered. For 66 patients, no histological material was available and 65 of these patients were considered disease-free, as there was no suspicion of malignancy at the initial workup or during a follow-up of at least 6 months either. One patient was diagnosed with a locally advanced bladder tumour on imaging findings and malignant urothelial cells were identified by cytology, but no further investigations were carried out due to the patient’s age and performance status.

Table 2 illustrates the sensitivities, specificities, positive and negative predictive values of urinary cytology, cystoscopy and the *UCA1* test designed to detect BC in the screening and follow-up groups.

**Table 2:** Sensitivity of diagnostic methods for bladder cancer (including *carcinoma* in situ) according to grade and stage

		*UCA1* test		Cytology		Cystoscopy	
Overall	n = 69						
Urothelial carcinoma	n = 65	46/65	70.8%	29/58^a^	50%	51/63^d^	81%
Low-grade	n = 23	14/23	60.9%	2/17	11.8%	19/23	82.6%
High-grade	n = 35	25/35	71.4%	24/34	70.6%	29/34	85.3%
CIS	n = 7	7/7	100%	3/7	42.9%	3/6	50%
Squamous cell carcinoma	n = 2	1/2		1/2		1/2	
Adenocarcinoma	n = 1	0/1		0/1		0/1	
Small cell carcinoma	n = 1	1/1		1/1		1/1	
pTa	n = 21	12/21	57.1%	2/15^b^	13.3%	18/21	85.7%
pT1	n = 15	14/15	93.3%	9/15	60%	13/15	86.7%
≥pT2	n = 26	15/26	57.7%	17/25^c^	68%	19/25^e^	76%

^a^ 7 cases were unsatisfactory for interpretation (6 low grade and 1 high grade tumors); ^b^ 6 cases were unsatisfactory for interpretation; ^c^ 1 case was unsatisfactory for interpretation; ^d^ cystoscopy was not performed in 2 cases (1 high grade tumors and 1 CIS); ^e^ cystoscopy was not performed in 1 case.

The overall sensitivities of cytology, cystoscopy and the *UCA1* test were 50.8, 79.1 and 70%. The sensitivity of the *UCA1* test was higher in the screening group compared to the follow-up group, (83.9 vs. 59%), yielding a negative predictive value of 86.1 and 68% respectively. Cystoscopy and urinary cytology were also less sensitive for detecting recurrences, with cystoscopy remaining the most efficient procedure (90 and 70.3% sensitivity for screening and follow-up), while urinary cytology detected 71.4% of primary BCs and only 34.3% of recurrences. Conversely, the *UCA1* test proved to be more specific in the follow-up group as compared to the screening group (77.3 vs. 64.6%), yielding a positive predictive value of 64.5% overall. The specificity of cystoscopy in the follow-up group was equivalent (77.3%) to the *UCA1* test, but both cystoscopy and cytology were more specific than the *UCA1* test in the screening setting.

Table 3 illustrates the number and percentages of patients reported as positive by cytology, cystoscopy and the *UCA1* test according to grade and stage. Cytology was more often positive in BC of higher grade (70.6% in high-grade vs. 11.8% in low-grade tumours) and stage (13.3% for pTa, 60% for pT1 and 71.4% for ≥pT2 tumours). Conversely, the result of the *UCA1* test did not vary significantly between low-grade and high-grade tumours (60.9 vs. 71.4%) and did not correlate with stage (57.1% for pTa tumours vs. 93.3% for pT1 tumours; 59.1% for ≥pT2 tumours). Cystoscopy remained the most efficient diagnostic tool, irrespective of grade and stage, except for CIS. Interestingly, the *UCA1* test successfully detected all seven cases of CIS and was more sensitive than cytology or cystoscopy in this setting. The *UCA1* test was also positive in one of two cases of squamous cell carcinoma and small cell carcinoma; however, the only case of primary bladder adenocarcinoma did not express *UCA1*.

**Table 3:** Diagnostic value of the *UCA1* test, urinary cytology and cystoscopy for the detection of bladder cancer

	*UCA1* test	Cytology	Cystoscopy
Screening			
Sensitivity	83.9%	71.4%	90%
Specificity	64.6%	97.6%	90%
Negative predictive value	86.1%	83.7%	92.3%
Positive predictive value	60.5%	95.2%	87.1
Follow-up			
Sensitivity	59%	34.3%	70.3%
Specificity	77.3%	94.7%	77.3%
Negative predictive value	68%	61%	75.6%
Positive predictive value	69.7%	85.7%	72.2%
Overall			
Sensitivity	70%	50.8%	79.1%
Specificity	70.7%	96.3%	83.3%
Negative predictive value	75.6%	71.3%	83.3%
Positive predictive value	64.5%	91.4%	79.1%

## Discussion

Since its first description in 2006, several studies have confirmed *UCA1* to be a biomarker for urothelial carcinoma and studied its expression in other cancers, including colorectal and breast cancers (Wang et al. 2006, 2008, 2012; Yang et al. 2012; Zhang et al. 2012; Srivastava et al. 2014; Han et al. 2014; Huang et al. 2014). Moreover, long non-coding RNAs, like *UCA1*, are increasingly thought to play a pivotal role in cancer development and progression (Shi et al. 2013; Li and Chen 2013). To date, only three studies have addressed the role of *UCA1* as a urinary marker. BC is the second most common urologic malignancy, yet it is one of the most challenging to treat due to significant tumour heterogeneity and potential life-long follow-up, which includes time-consuming and invasive procedures. The implementation of a urinary biomarker in clinical practice could allow for longer intervals between cystoscopies and a less invasive follow-up, but should not come at the expense of lower sensitivity.

In previous studies, the sensitivity of the *UCA1* marker ranged from 79.5 to 88.5%. With an overall sensitivity of 70%, the ability of the *UCA1* test to detect BC was significantly lower in our series. However, when limited to the screening group, the *UCA1* test yielded a sensitivity of 83.9%, which is comparable to reported results. Two of the previous studies did not detail the patients’ history of BC, but Srivastava et al. reported a significant difference in expression of *UCA1* in recurrent tumours compared to primary tumours (p < 0.001), emphasizing the necessity to analyse patients with recurrent BC separately when evaluating a novel biomarker (Srivastava et al. 2014). The positive and negative predictive values of the *UCA1* test were lower than previously reported (64.5 and 75.6% respectively), despite a relatively high prevalence of bladder cancer in our patients population (Srivastava et al. 2014).

The overall specificity of the *UCA1* test was 70.7%, ranging lower than the previously published results (79.7–92.3%). In our study, 27 patients with a positive *UCA1* test had no evidence of disease for at least 6 months and up to 53 months of follow-up. Nevertheless, late recurrences have been reported, which provide reasonable explanations for false positive results with other urinary markers, particularly FISH-based markers (Kamat et al. 2014 Oct). Anticipatory false-positive findings cannot be excluded at this stage and overexpression of *UCA1* may be due to its expression in early precursor lesions that remain clinically undetectable. This hypothesis is supported by the detection of *UCA1* in all seven cases of CIS and, additionally, in two cases of urothelial dysplasia (data not shown). Though four patients with false-positive tests were under surveillance for BC due to exposure to aristocholic acid, and one patient had been diagnosed with chronic schistosomiasis—two conditions associated with urothelial carcinogenic ability and urothelial dysplasia –, we did not find any association between particular clinical features and a false-positive test (Botelho et al. 2011; Lemy et al. 2008). Our study included only seven patients with CIS, but it is interesting to note that the *UCA1* test detected three additional cases compared to cystoscopy. However, in order to evaluate the efficiency of the *UCA1* test in flat urothelial lesions, further studies on larger cohorts are necessary.

Contrary to previous reports where *UCA1* has emerged as a particularly sensitive marker for superficial high-grade tumours, we did not find any significant difference in terms of sensitivity when the *UCA1* test was analysed according to grade. Although the *UCA1* test was particularly efficient in detecting pT1 tumours, the sensitivity for both non-invasive and muscle-invasive tumours was 57%.

*UCA1* expression was increased in various urothelial neoplastic lesions, including urothelial dysplasia, CIS and papillary low-grade and high-grade urothelial carcinomas—each associated with different morphology, pathogenesis and prognostic implications. Increased expression of *UCA1* has been associated with tumour proliferation, migration and invasion, though the exact mechanisms are yet to be elucidated. Recently, Wang et al. reported that *UCA1* promotes cell growth by downregulating the cell cycle inhibitor p21 via BRG1, a chromatin remodelling factor with anti-tumour properties (Wang et al. 2014). Furthermore, upregulated *UCA1* has been shown to promote resistance to cisplatin-based chemotherapy in bladder cancer cells (Fan et al. 2014). Taken together, these findings indicate that *UCA1* plays an important role in BC pathogenesis and progression, while its characterisation may provide new insights in early stages of BC development and, possibly, even new prognostic factors and therapeutic targets. It remains to be proven whether *UCA1* is a suitable diagnostic urinary biomarker in BC and a useful contribution to already existing diagnostic procedures in daily urologic practice.

In conclusion, the efficiency of the *UCA1* test for the diagnosis of BC in our study was lower than previously reported. Our results highlight the importance of testing novel urinary biomarkers in specific patient populations. The *UCA1* test cannot replace cystoscopy for the evaluation of patients with suspected primary BC or in the context of a follow-up for bladder cancer. While it may aid in the detection of CIS, more extensive studies are needed to confirm these findings. Conversely, our results did not provide evidence that the *UCA1* test is suitable for the follow-up of patients with previous BC, due to its low sensitivity in this population.

## End note

The present study has been approved by the Ethical Committee of the Erasme University Hospital (Ref: P2010/338).
